# Hsp90 Inhibitors Inhibit the Entry of Herpes Simplex Virus 1 Into Neuron Cells by Regulating Cofilin-Mediated F-Actin Reorganization

**DOI:** 10.3389/fmicb.2021.799890

**Published:** 2022-01-10

**Authors:** Xiaowei Song, Yiliang Wang, Feng Li, Wenyan Cao, Qiongzhen Zeng, Shurong Qin, Zhaoyang Wang, Jiaoyan Jia, Ji Xiao, Xiao Hu, Kaisheng Liu, Yifei Wang, Zhe Ren

**Affiliations:** ^1^Guangzhou Jinan Biomedicine Research and Development Center, Institute of Biomedicine, College of Life Science and Technology, Jinan University, Guangzhou, China; ^2^Key Laboratory of Virology of Guangzhou, Jinan University, Guangzhou, China; ^3^Key Laboratory of Bioengineering Medicine of Guangdong Province, Jinan University, Guangzhou, China; ^4^College of Pharmacy, Jinan University, Guangzhou, China; ^5^The Second Clinical Medical College, Shenzhen People’s Hospital, Jinan University, Guangzhou, China

**Keywords:** herpes simplex virus 1, heat shock protein 90, virus entry, cofilin, F-actin

## Abstract

Herpes simplex virus 1 (HSV-1) is a common neurotropic virus, the herpes simplex encephalitis (HSE) caused by which is considered to be the most common sporadic but fatal encephalitis. Traditional antiviral drugs against HSV-1 are limited to nucleoside analogs targeting viral factors. Inhibition of heat shock protein 90 (Hsp90) has potent anti-HSV-1 activities *via* numerous mechanisms, but the effects of Hsp90 inhibitors on HSV-1 infection in neuronal cells, especially in the phase of virus entry, are still unknown. In this study, we aimed to investigate the effects of the Hsp90 inhibitors on HSV-1 infection of neuronal cells. Interestingly, we found that Hsp90 inhibitors promoted viral adsorption but inhibited subsequent penetration in neuronal cell lines and primary neurons, which jointly confers the antiviral activity of the Hsp90 inhibitors. Mechanically, Hsp90 inhibitors mainly impaired the interaction between Hsp90 and cofilin, resulting in reduced cofilin membrane distribution, which led to F-actin polymerization to promote viral attachment. However, excessive polymerization of F-actin inhibited subsequent viral penetration. Consequently, unidirectional F-actin polymerization limits the entry of HSV-1 virions into neuron cells. Our research extended the molecular mechanism of Hsp90 in HSV-1 infection in neuron cells and provided a theoretical basis for developing antiviral drugs targeting Hsp90.

## Introduction

Herpes simplex virus type 1 (HSV-1) is a neurotropic DNA virus belonging to the Herpesviridae family (Grinde, 2013). The infection of HSV-1 is extensive and causes disease diversity. In particular, HSV-1, as a neurotropic virus, spreads to the brain. After primary infection, HSV-1 can reach the central nervous system, resulting in substantial damage to neuronal cells (Wang et al., 2019; Marcocci et al., 2020). Herpes simplex encephalitis (HSE) induced by HSV-1 infection is the most common sporadic but fatal encephalitis. Although rare, HSE causes 70% mortality in untreated patients (Whitley, 2006; Stahl et al., 2012; Piret and Boivin, 2020), and most survivors present severe neurological sequelae (Lafaille et al., 2019). Further research indicates that HSV-1 infection of neurons activates neurotoxic pathways typical of Alzheimer’s disease (AD), and repeated HSV-1 reactivations in the brain of infected mice produce an AD-like phenotype. HSV-1 is gradually recognized as an important factor affecting AD development (Balin and Hudson, 2018; Eimer et al., 2018; Tzeng et al., 2018; Wang et al., 2020b).

The proliferation process of HSV-1 includes multiple stages (Ibáñez et al., 2018). Traditional antiviral drugs against HSV-1 are limited to nucleoside analogs targeting viral factors, and the treatment drugs are single; thus, novel therapeutic targets against HSV-1 infection-associated diseases are urgently needed (Wang et al., 2017). Virus propagation requires host proteins to participate; to find new antiviral targets from host proteins is an appropriate strategy for developing safe and effective anti-herpes simplex drugs (Wang et al., 2017). The heat shock protein 90 (Hsp90) as a molecular chaperone associates with various other proteins such as steroid receptors, protein kinases, and filamentous actin and activates or inhibits a range of client proteins (Buchner and Li, 2013; Schopf et al., 2017). Hsp90 is essential for many viruses to complete the multiplication cycle, including human immunodeficiency virus (HIV)-1, enterovirus 71 (EV71), HSV-1, and influenza virus (Wang et al., 2017). Recent studies have indicated that Hsp90 is a crucial host factor that is required by many viruses for multiple phases of their life cycle, such as virus entry and intracellular transport, viral gene expression, and viral protein maturation which requires Hsp90 regulation (Wang et al., 2017; Li et al., 2018). Hsp90 has attracted intensive attention in developing novel antiviral drugs because of their functions in the life cycle of multiple viruses. For example, Hsp90 inhibitors block HSV-1 nuclear egress and assembly in Vero cells (Ju et al., 2011; Xiang et al., 2012a; Li et al., 2018), and another research shows that Hsp90 is essential for the correct localization of HSV-1 DNA polymerase to the nucleus (Burch and Weller, 2005), and Hsp90 also promotes nuclear transport of HSV-1 capsid proteins by interacting with acetylated tubulin (Zhong et al., 2014). Furthermore, previous research showed that AT533 reduced the nuclear translocation of NF-κB and NLRP3 activation *via* inhibiting Hsp90 from attenuating HSV-1-induced inflammation (Li et al., 2019b). Hsp90 inhibitors have rarely reported their antiviral effects in neurons, and it is of great significance to study Hsp90 as a promising host target for antiviral infection in neurons.

Viral intracellular transportation mainly relies on the dynamic regulation of the cell’s cytoskeleton, especially actin filaments, an important part of the cell cytoskeleton, which is closely related to early virus infection (Xiang et al., 2012b). Actin filaments are polymers formed by G-actin polymerization, mainly in the cortical area below the cell membrane (Sens and Plastino, 2015); F-actin dynamics is essential for early virus infection (Xiang et al., 2012b). Rho GTPase is a key regulator for regulating the actin dynamics, which is also closely related to viral infections (Favoreel et al., 2007; Van den Broeke et al., 2014). For example, the actin-depolymerizing factor (ADF)/cofilin regulates the microfilament framework (Favoreel et al., 2007; Ndozangue-Touriguine et al., 2008). Cofilin is essential for regulators of actin dynamics, is previously reported to sever actin filaments at low cofilin/actin ratios, and stabilizes filaments at high cofilin/actin ratios in mammalian neurons by binding to ADP–actin subunits in F-actin (Southwick, 2000; Bravo-Cordero et al., 2013; Wioland et al., 2017). When various intracellular and extracellular signal molecules stimulate cells, the microfilament can form filopodia, lamellipodia, and stress fibers (Taylor et al., 2011).

As an essential facet of virus–cell interactions, HSV interacts with the host cytoskeleton at various stages of the viral life cycle. The biphasic F-actin dynamics in HSV-1 neurons was demonstrated (Xiang et al., 2012b; Zheng et al., 2016). For example, F-actin dynamics plays a vital role in virus entry; we have shown that early infection of HSV-1 mobilizes F-actin reorganization to facilitate viral entry (Xiang et al., 2012b). F-Actin is polymerized to form pseudopods, which enrich the receptors required by the virus and facilitate further infection of the virus (Hu et al., 2016; Azab and Osterrieder, 2017), and previous studies have shown that the viral envelope glycoprotein gB binds to the receptor α3β1, activates Rho GTPases to cause the rearrangement of F-actin, and ultimately promotes virus entry into the cell (Naranatt et al., 2003). The entry of the virus into host cells, including adsorption and penetration, is the initial step of the infection process and is an ideal antiviral stage (Ibáñez et al., 2018). It is a good antiviral strategy to block the assembly of G-actin by regulating the polymerization and depolymerization of F-actin to inhibit virus entry (Zheng et al., 2016). Hsp90 is associated with filamentous actin (Kellermayer and Csermely, 1995; Taiyab and Rao, 2011), but the specific mechanism of Hsp90 regulating filamentous actin remains to be further explored. Herein, we further investigated the antiviral efficacy and mechanism of Hsp90 inhibitors in neuron cells and provided a theoretical basis for Hsp90 as a new anti-HSV-1 drug target point in HSE.

## Materials and Methods

### Cell and Virus

A Vero cell line (ATCC, Manassas, VA, United States) was cultured as previously described (Li et al., 2019a), The neuroblastoma cell line SH-SY5Y (ATCC, United States) was purchased from the Cell Bank of the Chinese Academy of Sciences, and N2a cells, our laboratory stock, were cultured in Dulbecco’s modified Eagle’s medium (DMEM; Gibco, Grand Island, NY, United States) with 10% fetal bovine serum (FBS; Gibco). The reporter virus EGFP-HSV-1, which expresses eGFP fused with US11, was acquired from the Anti-Stress and Health Research Center, College of Pharmacy, Jinan University, Guangzhou, Guangdong, 510632, China. HSV-1 strain F was obtained from Hong Kong University and preserved in our laboratory elaborately. After repeated freezing and thawing three times, the viruses were propagated in Vero cells, centrifuged at 4°C, 1,000 rpm for 5 min to take the supernatant, and then stored at –80°C until used.

### Chemicals, Antibodies, and Reagents

17-N-Allylamino-17-demethoxygeldanamycin (17-AAG) was purchased from Selleck (S1141; Houston, TX, United States) and dissolved in dimethyl sulfoxide at a concentration of 10 mM. SNX-25a (AT533) was acquired according to previous reports [20] and dissolved in dimethyl sulfoxide at a concentration of 20 mM. In this study, SH-SY5Y cells were treated with 17-AAG (1.25 μM) or AT533 (1.00 μM), and N2a cells were treated with 17-AAG (0.63 μM) or AT533 (40.00 nM). Cytochalasin B was purchased from Sigma-Aldrich (14930-96-2, St. Louis, MO, United States). Antibodies, including anti-gB (ab6506) and anti-VP5 (ab6508), were purchased from Abcam (Cambridge, United Kingdom), as well as anti-cofilin (5175S), anti-p-cofilin (3313 S), anti-Hsp90 (ab13492), anti-Hsp90α (GTX109753), anti-Hsp90β (5087 S), anti-β-actin (GTX109639), and anti-GAPDH (5174 S). TRIzol reagent was bought from Tiangen (Beijing, China). All primer sequences are shown in Supplementary Table 1. siRNAs were purchased from GenePharma (Shanghai, China), and their sequences are shown in Supplementary Table 2.

### CCK8 Assay

The cytotoxicity of 17-AAG or AT533 on SH-SY5Y and N2a cells was evaluated by CCK8 assay. Both SH-SY5Y and N2a cells were seeded into 96-well plates to 90% confluence; then, various concentrations of the compound were added to the plate. After 24 h, each well was added with 10 μl of CCK8 solution and incubated at 37°C for another 2 h in the dark. The absorbance was then measured by an enzyme immunoassay reader at 450 nm.

### Quantitative Real-Time PCR

The total RNA of the different cell samples was extracted according to the manufacturer’s specifications (TRIzol, DP424, Tiangen Biotech Co., Beijing, China) and was reverse transcribed using the Reverse Transcriptase Kit (RR036A-1, TaKaRa, Tokyo, Japan). Quantitative real-time PCR (qRT-PCR) was performed using a Bio-Rad CFX96 real-time PCR system with a total volume of 10 μl including 5 μl of SYBR Green Real-Time PCR Master Mix (RR820, TaKaRa, Japan), 4 μl of cDNA template, and 0.5 μl of each primer. Data were processed and analyzed by Bio-Rad CFX Manager software. The mRNA expression normalization was internally controlled by gene β-actin.

### Western Blotting

Cell samples were washed thrice with precooled PBS and lysed in SDS buffer (Beyotime, Shanghai, China) containing 1 mM PMSF (Beyotime). Lysates were heated at 100°C for 15 min and centrifuged at 12,000 × g for 15 min at 4°C. The protein concentrations were then measured with a bicinchoninic acid (BCA) protein assay kit (Beyotime), and the cell lysates were mixed with 5 × SDS-PAGE buffer (P0015, Beyotime, Shanghai, China) and boiled for 10 min. Subsequently, samples were subjected to 10–12% SDS-PAGE and transferred to a polyvinylidene fluoride (PVDF) membrane (Millipore, Bedford, MA, United States). The PVDF membranes were incubated with interesting primary antibodies overnight at 4°C after blocking with 5% skimmed milk for 1 h, then indicated with appropriate HRP-conjugated secondary antibodies (Invitrogen, Carlsbad, CA, United States) for 1 h at room temperature. Finally, target proteins were visualized by enhanced chemiluminescence.

### Detection of Herpes Simplex Virus 1 Binding and Internalization

The amount of virus binding to and internalized by cells was measured as described previously, just with minor modifications (Wang et al., 2020a). Cells were seeded 24 h prior to the experiment in 12-well plates at a 2 × 105/well density to determine the amount of virus binding. Cells were incubated for 1 h at 4°C, then infected with HSV-1 for another 1 h in the presence of 17-AAG or AT533. The unbound virus was removed with PBS after HSV-1 attachment. The total DNA of HSV-1 was extracted using the EasyPure^®^ Viral DNA/RNA Kit (Trans, Beijing, China) to determine viral DNA copy numbers using qRT-PCR-based detection of the viral gene. To measure the amount of viral entry, cells were seeded 24 h prior to the experiment in 12-well plates at a 2 × 10^5^/well density. Cells were incubated for 1 h at 4°C, then infected with HSV-1 for another 1 h, and then washed with PBS for three times before the cells were transferred to 37°C until the end of penetration; the HSV-1 internalized by cells was detected after extracellular viral particles and virions that bound to but did not enter the cells were removed by washing with PBS (pH 3.0). The cells were collected, and total DNA was extracted by EasyPure Viral DNA/RNA Kit, and targeted genes were analyzed by qRT-PCR. In addition, other experiments were performed on these cell samples according to the experimental objectives.

### Viral Plaque Assay

SH-SY5Y and Vero cells were seeded in 24-well plates at a density of 1.5 × 105/well, then the reduction of plaque formation was measured. Briefly, cells were incubated for 1 h at 4°C, then infected with HSV-1 for another 1 h. The virus inoculum was removed, and an overlay medium (maintenance medium containing 1% methylcellulose) was added to each well. After 72 h of incubation, cell monolayers were fixed with 4% paraformaldehyde and stained with 1% crystal violet. Finally, the plaque number was counted.

### Immunofluorescence Assay

Cells were seeded into confocal dishes to attain 70–80% confluence and treated based on various experimental requirements, and then samples were fixed with 4% paraformaldehyde for 20 min, permeabilized with 0.1% Triton X-100 for 5 min, and then blocked with 5% bovine serum albumin for 1 h. The samples were incubated overnight at 4°C with the anti-VP5 primary antibody then incubated with a fluorescently labeled secondary antibody for 1 h at room temperature, then actin filaments were stained by FITC-labeled phalloidin (red), and cell nuclei were stained with DAPI staining solution (C1006, Beyotime, Shanghai, China) for 10 min. Finally, a confocal laser scanning microscope was used to capture immunofluorescence images (LSM 510 meta; Zeiss).

### Immunoprecipitation Assay

Cells were lysed with 500 μl IP lysis buffer (P0013J, Beyotime, Shanghai, China) containing 1% PMSF, and the supernatant was collected by centrifugation at 14,000 g for 10 min at 4°C. Fifty microliters of supernatant was used as input, and the remaining supernatant was incubated with a primary antibody (Hsp90, cofilin) overnight at 4°C. Then, 40 μl of Protein A/G magnetic beads were incubated for 4 h on a vertical suspension at 4°C. The immunoprecipitation was collected and washed three times with ice PBS and resuspended in 30 μl of 1 × SDS-PAGE buffer. Finally, the sample was analyzed by Western blot.

### Flow Cytometry

Cells were infected with HSV-1 and treated with 17-AAG, AT533, or cytochalasin B, and the samples were processed accordingly according to the purpose of the experiment. Then, the cells were washed with PBS, fixed with 4% paraformaldehyde for 5 min, and permeabilized with 0.1% Triton X-100 for 5 min. Next, all samples were stained with 5% FITC phalloidin (40735ES75, YEASEN, China) for 40 min at 37°C. Finally, fluorescence was analyzed with a flow cytometer (Becton Dickinson, San Jose, CA, United States).

### Primary Cortical Neuron Extraction

The extraction of cortical neurons was measured as described previously (Meng et al., 2017). We extracted cortical neurons from C57BL/6J mice 1–2 days after birth. For example, 0.1 mg/ml L-polylysine (P8130-25, Solarbio, Beijing, China) was coated on a six-well plate overnight, washed with ultrapure water, and exposed to ultraviolet light in a biological safety cabinet and dried. C57BL/6J mice were sterilized with 75% alcohol, and the cerebral cortex was taken out and cut into pieces in a container of 2 ml D-Hanks solution, then 1 ml of 0.25% trypsin was added for digestion at 37°C, 5% CO_2_ for 10 min. The tissue suspension was filtered with a primary cell strainer, and the cells were collected at 1,000 rpm, centrifuged for 5 min, and seeded in a six-well plate at 1.5 × 10^6^ cells/well. Finally, the cells were cultured in a 37°C, 5% CO_2_ incubator for 6 h, and then the adhesion of the cells was observed. After most of the cells have adhered, the medium was changed and a freshly prepared Neurobasal-A + B27 medium was added. The second day of replacing the Neurobasal-A + B27 medium was used as the first day of *in vitro* cell culture. On the third day, the Neurobasal-A + B27 medium was changed again. On the fifth day, neurons were separated and subjected to corresponding experiments.

### Statistical Analysis

Data were presented as mean ± SD of the results from at least two independent experiments. Significant differences between control and experimental groups were analyzed using Student’s *t*-test. Differences were considered significant set at *p < 0.05. ^**^p < 0.01; ^***^p < 0.001.

## Results

### Hsp90 Inhibitors Inhibit Herpes Simplex Virus 1 Infection of Neuron Cells

**FIGURE 1:**
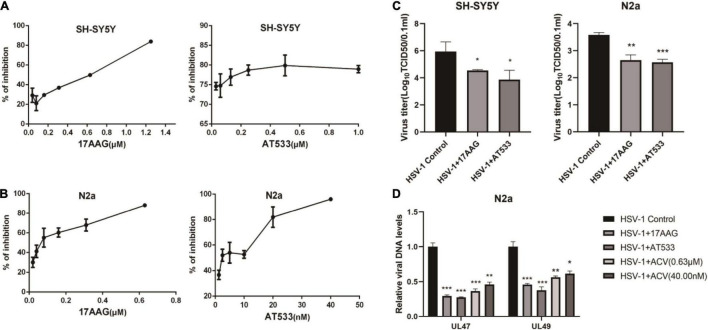
The antiviral activity of Hsp90 inhibitors. **(A,B)** Hsp90 inhibitors exhibited an inhibitory effect on eGFP-HSV-1. SH-SY5Y and N2a cells were infected with eGFP-HSV-1 (MOI = 0.1) at different concentrations of 17-AAG or AT533 for 24 h. The fluorescence intensity was detected by a multifunctional enzyme marking instrument to calculate the virus inhibition rate. **(C)** SH-SY5Y and N2a cells were infected with HSV-1 (MOI = 0.1) in 17-AAG or AT533 for 24 h, and the cell suspensions were collected to measure the viral titer in Vero cells. **(D)** N2a cells were infected with HSV-1 (MOI = 0.1) in the presence of 17-AAG or AT533 for 24 h, and ACV was used as a positive control, then the total DNA of HSV-1 was extracted, and the levels of *UL47* and *UL49* were analyzed by qRT-PCR. Data are mean ± SD (*n* = 3). *p < 0.05, **p < 0.01, ***p < 0.001 versus the HSV-1 control group.

Two different Hsp90 inhibitors, 17-AAG and AT533, were used to detect cytotoxicity and antiviral activity. Firstly, the cytotoxicity of Hsp90 inhibitors on SH-SY5Y and N2a cells was detected by CCK8 assay (Supplementary Figures 1A,B). In addition, we used eGFP-HSV-1 to infect SH-SY5Y and N2a cells for 24 h in the presence of Hsp90 inhibitors, respectively, and the antiviral activity of Hsp90 inhibitors was judged by the fluorescence intensity of eGFP (Figures 1A,B). Furthermore, we also assessed the effects of Hsp90 inhibitors on viral titers (Figure 1C), which indicated that 17-AAG and AT533 inhibited HSV-1 infection. HSV-1 DNA was also extracted by the Virus DNA/RNA Kit to detect the amount of HSV-1. The results show that 17-AAG and AT533 have better antiviral activity than ACV (Acyclovir) (Figure 1D).

### Hsp90 Inhibitors Promote Herpes Simplex Virus 1 Attachment

We consider that the adsorption of HSV-1 on the cell membrane surface is the first step in the virus proliferation process (Ibáñez et al., 2018). To explore the effect of Hsp90 inhibitors on the adsorption phase of HSV-1-infected neuron cells, two different Hsp90 inhibitors, 17-AAG and AT533, were treated as neuron cells. A simple diagram of HSV-1 attachment was shown (Supplementary Figure 2A). We firstly investigated the amount of HSV-1 bound to cells by quantifying the viral genome, a common method used for determining the amount of viral attachment during early infection (Wang et al., 2020a). Neuron cells are pretreated at 4°C for 1 h and then infected with HSV-1 1 h at 4°C, so that the virus can be infected simultaneously as much as possible, after the adsorption is completed. Hsp90 inhibitors were treated on the adsorption of HSV-1-infected SH-SY5Y and N2a cells, then HSV-1 DNA was extracted by the Virus DNA/RNA Kit for detection by qRT-PCR.

**FIGURE 2:**
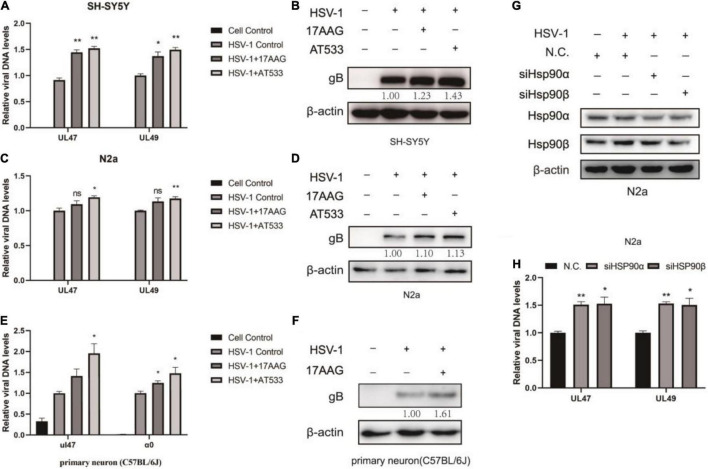
Hsp90 inhibitors promote HSV-1 attachment. **(A)** Hsp90 inhibitors increased the amount of viral DNA. SH-SY5Y cells were incubated for 1 h at 4°C, then infected with HSV-1 (MOI = 20) for another 1 h in the presence of 17-AAG or AT533, and the unbound virus was removed after HSV-1 attachment. Total DNA of HSV-1 was extracted, and the expression level of *UL47* and *UL49* was subjected to qRT-PCR analysis. **(B)** Hsp90 inhibitors increased the amount of viral protein. SH-SY5Y cells were incubated for 1 h at 4°C, then infected with HSV-1 (MOI = 50) for another 1 h in the presence of 17-AAG or AT533, and the unbound virus was removed after HSV-1 attachment. The level of gB was detected by Western blot. **(C,D)** N2a cells were treated as **(A,B)** in the presence of Hsp90 inhibitors, and the total DNA and protein of HSV-1 were extracted for analysis. **(E)** Hsp90 inhibitors promoted the amount of viral DNA in primary cortical neurons. Primary cortical neurons were incubated for 1 h at 4°C, then infected with HSV-1 (MOI = 20) for another 1 h in the presence of 17-AAG or AT533, then viral DNA was extracted and then qRT-PCR was performed to measure viral DNA amounts. **(F)** 17-AAG promoted the amount of protein. Primary cortical neurons were incubated for 1 h at 4°C, then infected with HSV-1 (MOI = 50) for another 1 h in the presence of 17-AAG or AT533, the total proteins were extracted after HSV-1 attachment; protein levels were evaluated by Western blotting. **(G)** The efficiency of Hsp90α and Hsp90β knockdown was detected by Western blot for 48 h. **(H)** Hsp90α and Hsp90β knockdown also increased the level of HSV-1. Data are mean ± SD (*n* = 3). *p < 0.05, **p < 0.01 versus the HSV-1 control group.

Interestingly, the results indicated that viral attachment was higher than control groups after Hsp90 inhibitors treatment (Figures 2A,C). Similarly, Hsp90 inhibitors pretreatment also promoted HSV-1 attachment (Supplementary Figure 3). The level of gB, a viral protein carried by the virus particle itself, was increased than the virus control after the cells were treated with Hsp90 inhibitors (Figures 2B,D). Consistently, we also verified primary cortical neurons, and the cortical neurons of C57BL/6J mice were extracted as described previously (Meng et al., 2017). Cell morphology was shown (Supplementary Figure 4A), beyond the neuronal nuclear antigen (NeuN), a neuronal specific nuclear protein in vertebrates, as a neuronal nuclear antigen marker was detected, and the primary cortical neurons were separated with a purity of 95.76% (Supplementary Figure 4B). The DNA and protein levels were higher than the control group (Figures 2E,F). These results confirmed that Hsp90 inhibitors facilitated the adsorption of HSV-1 on neuron cells.

Hsp90α and Hsp90β are mainly involved in the virus proliferation cycle (Wang et al., 2017). Moreover, the knockdown efficiency was detected after Hsp90α and Hsp90β were knocked down by siRNA (Figure 2G). Samples were collected to extract viral DNA to determine the HSV-1 binding amount according to the “Detection of HSV-1 binding and internalization” method, and Hsp90α and Hsp90β knockdown promoted viral attachment (Figure 2H).

### Hsp90 Inhibitors Inhibit Herpes Simplex Virus 1 Penetration

**FIGURE 3:**
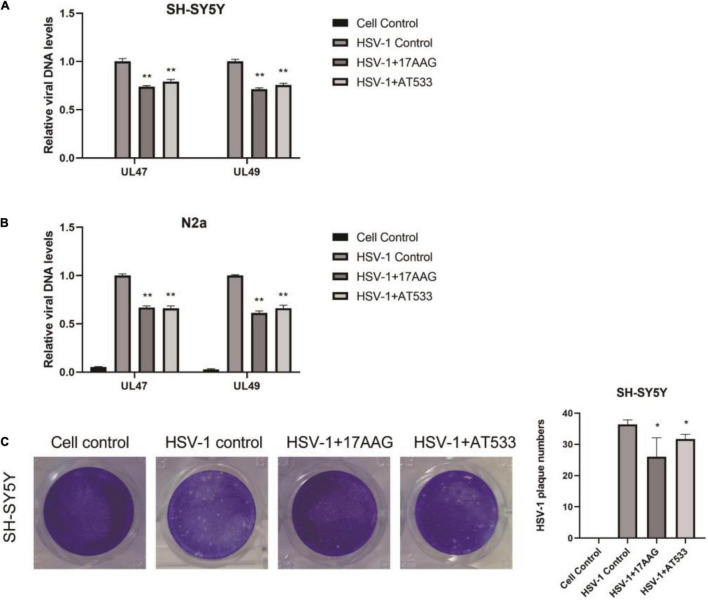
Hsp90 inhibitors abrogate HSV-1 penetration. **(A,B)** 17-AAG and AT533 reduced the amount of viral DNA. SH-SY5Y and N2a cells were precooled for 1 h at 4°C, then infected with HSV-1 (MOI = 20) for another 1 h at 4°C. The unadsorbed viruses were removed, then SH-SY5Y cells were treated with 17-AAG or AT533 for 0.5 h at 37°C and washed with acidic PBS to remove the viruses on cell membranes. Total DNA of HSV-1 was extracted for analysis. **(C)** HSV-1 infection-mediated plaque formation units were significantly reduced. 17-AAG and AT533 processed the penetration stage, then HSV-1 plaque formation was measured after 72 h post-infection in SH-SY5Y cells. Data are mean ± SD (*n* = 3). *p < 0.05, **p < 0.01 versus the HSV-1 control group.

Furthermore, Hsp90 is involved in multiple phases of the virus infection cycle (Wang et al., 2017). Thus, we further explored the effect of Hsp90 inhibitors on HSV-1 penetration, and a simple diagram of HSV-1 penetration was shown (Supplementary Figure 2B). The dynamic curve of HSV-1 penetration was tested (Supplementary Figures 5A,B). Firstly, neuronal cells were infected with HSV-1 for 1 h at 4°C; we found that the intracellular virus reached a plateau after 30 min of virus infection at 37°C, indicating the end of viral penetration. To further confirm the effects of Hsp90 inhibitors on HSV-1 early infection, the internalized viral DNA was quantified using real-time PCR. As shown in Figures 3A,B, Hsp90 inhibitors inhibit the amount of intracellular virus DNA. Antiviral activity was evaluated by a plaque reduction assay (PRA); the amount of HSV-1 infection-mediated plaque formation units was also significantly reduced by Hsp90 inhibitors (Figure 3C). Therefore, the above experiment indicates that Hsp90 inhibitors inhibit virus penetration.

### Hsp90 Inhibitors Inhibit Herpes Simplex Virus 1 Entry

**FIGURE 4:**
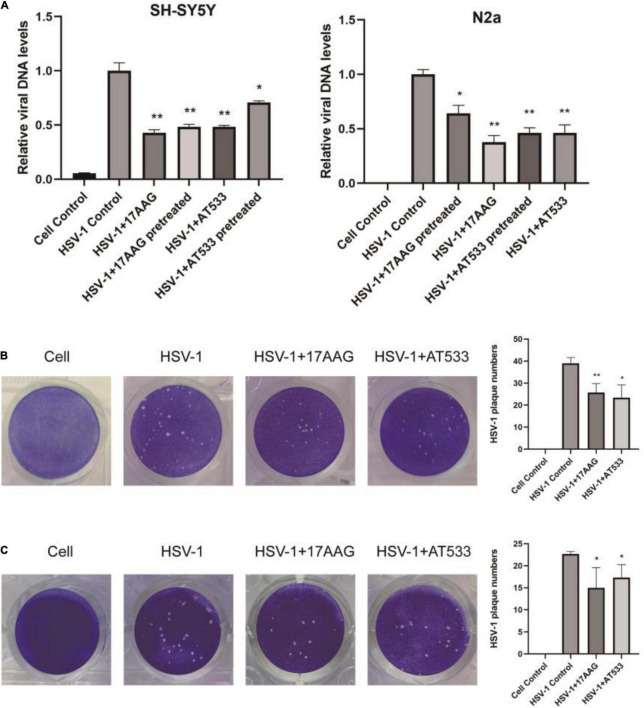
Hsp90 inhibitors block viral entry. **(A)** SH-SY5Y and N2a cells were pretreated with 17-AAG or AT533 for 1 h at 4°C and washed with PBS to remove the inhibitors, then infected with HSV-1 (MOI = 20) for another 1 h at 4°C, then all groups were shifted to 37°C; at the same time, the treatment groups were treated with HSV-1 (MOI = 20) in the presence of Hsp90 inhibitors all the time. Finally, viral DNA was extracted, and then qRT-PCR was performed to measure viral α*0* DNA amounts. **(B)** Plaque-formation assay results showing that Hsp90 inhibitors limited HSV-1 entry; SH-SY5Y cells were incubated for 1 h at 4°C, then infected with HSV-1 (MOI = 20) for another 1 h at 4°C in the presence of Hsp90 inhibitors, and all groups were shifted to 37°C, then washed with acidic PBS; overlay medium was added to each well. After 72 h of incubation, cell monolayers were fixed with 4% paraformaldehyde and stained with 1% crystal violet. Finally, the number of plaques was counted. **(C)** N2a cells were infected with HSV-1 (MOI = 20) in the presence of Hsp90 inhibitors, then unbound virus was removed, and N2a cells were treated with the Hsp90 inhibitor again; all groups were shifted to 37°C for 30 min, then all samples were repeatedly frozen and thawed three times to collect the virus, and the plaque reduction experiment was performed on Vero cells to evaluate the level of a virus entering N2a cells. Data are mean ± SD (*n* = 3). *p < 0.05, **p < 0.01 versus the HSV-1 control group.

Considering that the process of virus entry into host cells includes adsorption and penetration stages, the comprehensive effect of Hsp90 inhibitors on HSV-1 entry into neuron cells is worthy of further investigation. HSV-1 DNA was extracted using the viral DNA/RNA kit and detected by qRT-PCR. As shown in Figure 4A, whether it was pretreated and treated with 17-AAG or AT533, the entry of HSV-1 was inhibited. In addition, plaque reduction experiments also show that Hsp90 inhibitors impaired HSV-1 from entering SH-SY5Y cells (Figure 4B). While it is not suitable for N2a cells to directly form plaque, after HSV-1 (MOI = 20) infected N2a cells, the virus was therefore collected to infect Vero cells for plaque reduction experiments; consistently, Hsp90 inhibitor treatment significantly reduced the number of viral plaque (Figure 4C). These results further demonstrated that Hsp90 inhibitors played an antiviral effect at HSV-1 entry.

### Hsp90 Inhibitors Induce F-Actin Assembly

**FIGURE 5:**
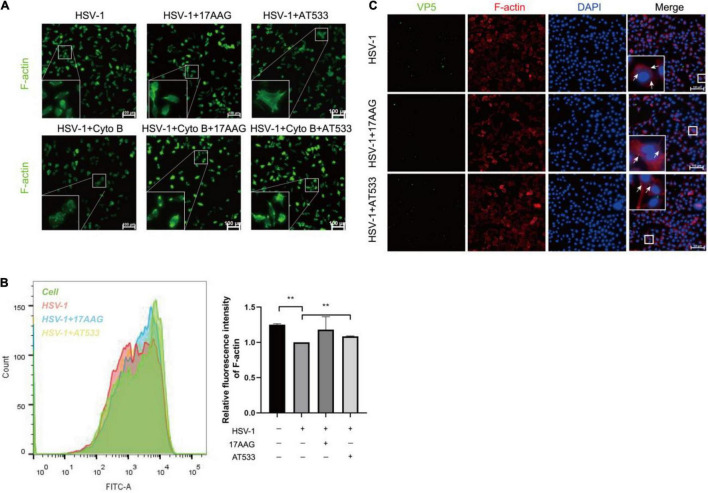
Hsp90 inhibitors promote the aggregation of F-actin. **(A)** Hsp90 inhibitors induced F-actin assembly in HSV-1 entry. SH-SY5Y cells were precooled for 1 h at 4°C then treated with 17-AAG, AT533, or Cyto B (10 μM) for 1 h at 4°C and another 0.5 h at 37°C, then the cells were collected to be fixed, and F-actin was stained with FITC-phalloidin (F-actin, green) for analysis by immunofluorescence assay. **(B)** Hsp90 inhibitors induced F-actin assembly in HSV-1 penetration. SH-SY5Y cells were incubated for 1 h at 4°C, then infected with HSV-1 (MOI = 100) for another 1 h at 4°C, then cells were treated with 17-AAG or AT533 for 30 min at 37°C. Then, F-actin was stained with FITC-phalloidin for analysis by flow cytometry. Data are mean ± SD (*n* = 2). **p < 0.01. **(C)** Hsp90 inhibitors impaired viral penetration by inducing F-actin assembly; SH-SY5Y cells were incubated for 1 h at 4°C, then infected with HSV-1 (MOI = 50) for another 1 h at 4°C, then cells were treated with 17-AAG or AT533 for 30 min at 37°C. The cells were then fixed and stained with anti-VP5 antibody (green), TRITC-phalloidin (F-actin, red), and DAPI (nucleus, blue), then analyzed by immunofluorescence assay. Scale bars, 100 μm. × 20.

Cell cytoskeleton, especially actin filaments, is an essential part of the cell structure, closely related to virus infection (Favoreel et al., 2007; Pollard and Cooper, 2009; Zheng et al., 2016; Miranda-Saksena et al., 2018). In the previous study, F-actin dynamics plays a vital role in virus entry. We have shown that early infection of HSV-1 mobilizes F-actin reorganization to facilitate viral entry (Xiang et al., 2012b). Considering that HSV-1 modifies the actin filaments *via* cofilin to facilitate its infection of neurons, specifically, HSV-1 infection firstly enhances F-actin polymerization to promote virus binding, which is followed by continuous depolymerization of F-actin during the later stages to facilitate viral penetration (Xiang et al., 2012b; Zheng et al., 2014). It has been reported that disrupting the dynamics of F-actin is a good antiviral strategy. For example, amentoflavone, a polyphenol compound, disturbed cofilin-mediated F-actin assembly and significantly reduced HSV-1 infection (Li et al., 2019a). In addition, the disruption of F-actin dynamics by chemical inhibitors also significantly reduced the efficiency of early viral infection and intracellular HSV-1 replication (Pei et al., 2011; Xiang et al., 2012b; Spear et al., 2013; Yi et al., 2017). Thus, we firstly analyzed the possible effect of Hsp90 inhibitors on the F-actin of neuronal cells; cytochalasin B (Cyto B), an inhibitor of F-actin, was used to induce the depolymerization of existing actin filaments, and the results indicated that Hsp90 inhibitors promoted the polymerization of F-actin to prevent Cyto B-induced depolymerization with HSV-1 infection (Figure 5A). Next, F-actin was measured by flow cytometry as described previously (Li et al., 2019a). As shown in Figure 5B, Hsp90 inhibitors induced F-actin assembly in the presence of HSV-1 infection. Finally, the effect of Hsp90 inhibitors on F-actin with the stages of HSV-1 penetration was also analyzed (Figure 5C). These results indicated that Hsp90 inhibitors promoted F-actin polymerization to prevent HSV-1 from entering neuron cells.

### Hsp90 Inhibitors Lead to a Reduced Membrane Localization of Cofilin by Impairing Hsp90–Cofilin Interaction

**FIGURE 6:**
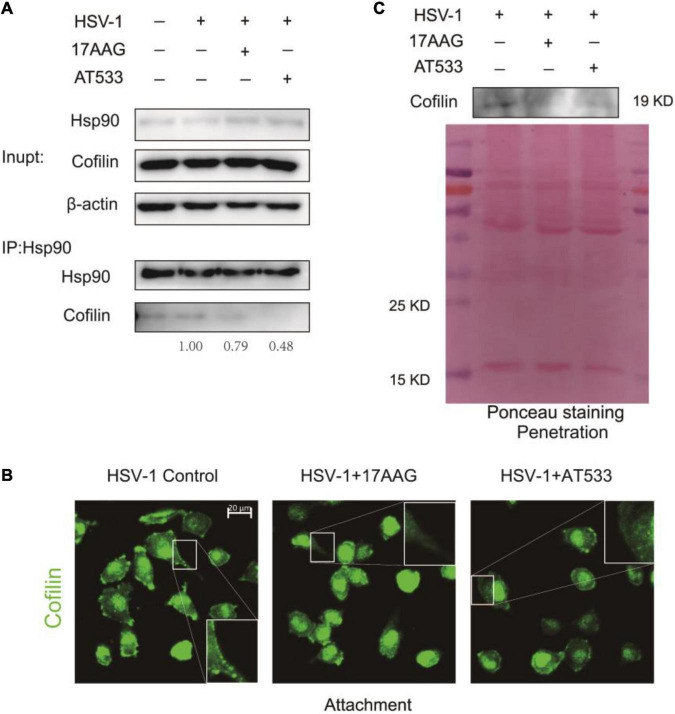
Hsp90 inhibitors reduce cofilin cell membrane distribution. **(A)** The binding of Hsp90 to cofilin was reduced in the presence of Hsp90 inhibitors, and the whole-cell extracts were subjected to immunoprecipitation with Protein A/G magnetic beads, anti-Hsp90, and anti-cofilin for immunoprecipitation assay, and the samples were detected by Western blot assay. **(B)** Hsp90 inhibitors affected the intracellular localization of cofilin; SH-SY5Y cells were incubated for 1 h at 4°C, then infected with HSV-1 (MOI = 50) at 4°C for another 1 h in the presence of 17-AAG or AT533. The cells were then fixed and stained with anti-cofilin antibody (green). Scale bars, 20 μm, ×40. **(C)** Hsp90 inhibitors reduced the localization of cofilin protein levels on the cell membrane. SH-SY5Y cells were incubated for 1 h at 4°C and infected with HSV-1 (MOI = 50) at 4°C for another 1 h; the unbound virus was removed, then treated with 17-AAG or AT533 for 30 min at 37°C, and cells were washed with acidic PBS. The cell membrane proteins were extracted to detect the protein level of cofilin by Western blotting. Data are mean ± SD (*n* = 3).

Given Hsp90 is a molecular chaperone for folding, assembling, and stabilizing many client proteins, which is essential for these client proteins to perform their normal biological functions (Bravo-Cordero et al., 2013; Balin and Hudson, 2018), inhibition of Hsp90 function may result in the degradation or abnormal subcellular localization of client proteins (Prodromou, 2012). To clarify the reason why Hsp90 inhibitors facilitated F-actin polymerization, we analyzed the effect of Hsp90 inhibitors on the ADF/cofilin; the results of co-immunoprecipitation indicated that Hsp90 interacted with cofilin, and Hsp90 inhibitors led to a weakened interaction of cofilin (Figure 6A). However, Western blot assay showed that Hsp90 inhibitors did not affect the total amount of cofilin protein in the stage of HSV-1 attachment (Supplementary Figures 6A,B) or HSV-1 penetration (Supplementary Figures 6C,D).

We further examined the effect of Hsp90 inhibitors on the subcellular localization of cofilin during the HSV-1 infection. Immunofluorescence showed that the Hsp90 inhibitor affected the intracellular localization of cofilin, resulting in a significant decrease in the localization of cofilin at the cell edge (Figure 6B). Considering that actin filaments are mainly located on the area under the cell membrane, and cofilin is also located on the cell membrane and combines with F-actin to play a depolymerization effect (Nebl et al., 1996; Keezer et al., 2003), we extracted the cell membrane proteins to detect the level of cofilin, and the results confirmed that Hsp90 inhibitors cause a decrease in the level of cofilin on the cell membrane during the penetration stage of HSV-1 (Figure 6C), which may lead to the polymerization of F-actin, thereby inhibiting the entry of HSV-1 into neuron cells.

## Discussion

HSE is the most common cause of sporadic fatal encephalitis worldwide (Whitley, 2006), and HSE shows the characteristics of high mortality and poor prognosis in all encephalitis, which is tightly associated with Herpes simplex virus 1 (HSV-1) infection of the neurons (Whitley et al., 1982; Whitley, 2006). Specifically, HSV-1 can spread from epithelial cells to neurons and cause pathological changes in the central nervous system (Wang et al., 2019). However, Hsp90 inhibitors have rarely reported their antiviral effects in neurons. This study demonstrated that Hsp90 inhibitors had antiviral activity against HSV-1 in nerve cell lines (Figure 1) and inhibited HSV-1 entry into neuronal cells (Figure 4). We consider that many viruses have evolved various methods of destroying the actin cytoskeleton to promote self-infection. For example, HIV and HSV-1 regulate the cofilin-mediated F-actin dynamic to facilitate virus entry (Yoder et al., 2008; Xiang et al., 2012b). The formation of pseudopods was beneficial to HSV-1 which adsorbs to the cell membrane, but the unidirectional polymerization of F-actin prevented HSV-1 penetration (Xiang et al., 2012b). We further examined the effect of Hsp90 inhibitors on ADF/cofilin. Specifically, both Western blotting assay and immunofluorescence experiments showed that Hsp90 inhibitors inhibited the molecular chaperone function of Hsp90 during HSV-1 infection, which reduced the subcellular localization of cofilin on the membrane to promote the assembly of actin structures (Figure 6). We hypothesize that the inhibition of HSV-1 penetration was a key rate-limiting step. Whether this phenomenon exists in non-neuronal cells remains to be further explored. It has been reported that geldanamycin, an Hsp90 inhibitor, has a significant inhibitory effect against HSV-1 in Vero cells (non-neural cells). Still, geldanamycin inhibits neither HSV-1 attachment nor penetration and mainly restrains HSV-1 replication *in vitro* (Li et al., 2004). However, in the enrichment of microfilament skeletons in synapses and dendritic spines in neuron cells, cofilin played an important role in the growth and development of nerves, including the formation of neurites, the directional growth of axons and dendrites, the formation of synapses, and the plasticity of neuronal cell dendritic spines (Sivadasan et al., 2016; Zheng et al., 2016; Tedeschi et al., 2019), which may be beneficial to Hsp90 inhibitors inhibiting the entry of HSV-1 into neuron cells by regulating cofilin-mediated F-actin reorganization.

In summary, this study demonstrated the effects of Hsp90 inhibitors 17-AAG and AT533 on HSV-1 infection of neuron cells. Specifically, we confirmed that Hsp90 inhibitors reduced membrane localization of cofilin by impairing Hsp90-cofilin interaction, which led to the unidirectional polymerization of F-actin. However, F-actin polymerization promoted viral attachment but inhibited subsequent viral penetration, which limited the entry of HSV-1 virions into neuron cells. Our research will provide a novel insight into the mechanism of Hsp90 inhibitors against HSV-1 and better extend the molecular mechanism of Hsp90 in HSV-1 infection in neuron cells.

## Data Availability Statement

The original contributions presented in the study are included in the article/Supplementary Material, further inquiries can be directed to the corresponding author/s.

## Ethics Statement

The animal study was reviewed and approved by Medical Ethics Committee of Jinan University.

## Author Contributions

XS, YLW, and FL conceived and designed the study. XS wrote the first draft of the manuscript. XS, WC, QZ, and JX developed and designed the methodology and specifically performed the experiments. SQ, ZW, JJ, and XH provided study materials. YLW commented or revised the manuscript. KL, ZR, and YFW were responsible for the management and coordination for the research activity planning and execution and acquisition of the financial support for the project leading to this publication. All authors have read and agreed to the published version of the manuscript.

## Conflict of Interest

The authors declare that the research was conducted in the absence of any commercial or financial relationships that could be construed as a potential conflict of interest.

## Publisher’s Note

All claims expressed in this article are solely those of the authors and do not necessarily represent those of their affiliated organizations, or those of the publisher, the editors and the reviewers. Any product that may be evaluated in this article, or claim that may be made by its manufacturer, is not guaranteed or endorsed by the publisher.

## Supplementary Material

Supplementary Figure 1 The cytotoxicity of Hsp90 inhibitors. **(A,B)** The cytotoxicity of Hsp90 inhibitors. SH-SY5Y and N2a cells were treated with different concentrations of 17AAG, AT533 for 24 h, and the cell survival was then calculated through CCK8 assay. Data are mean ± SD (*n* = 3).

Supplementary Figure 2 Simple diagram of HSV-1 attachment and penetration. **(A)** Cells were incubated for 1 h at 4°C, then infected with HSV-1 for another 1 h in the presence of 17AAG or AT533, and the unbound virus was removed after HSV-1 attachment **(B)** Cells were pre-cooled for 1 h at 4°C, then infected with HSV-1 for another 1h at 4°C. The unadsorbed viruses were removed, then the cells were treated with 17AAG or AT533 for indicated times at 37°C, and washed with acidic PBS to remove the viruses on cell membranes. All samples were collected to detect and analyze.

Supplementary Figure 3 Hsp90 inhibitors pretreatment promoted HSV-1 attachment. SH-SY5Y cells were pretreated with 17AAG or AT533 for 1 h at 4°C, and washed with PBS to remove the inhibitors, then infected with HSV-1 (MOI = 20) in the absence of 17AAG and AT533 for another 1 h. Total DNA of HSV-1 was extracted for qRT-PCR analysis to detect the level of *UL47* gene. Data are mean ± SD (*n* = 3). ***P* < 0.01 versus HSV-1 control group.

Supplementary Figure 4 The cell morphology of cortical neurons. **(A)** Primary cortical neurons were extracted from the cerebral cortex of C57BL/6J mice aged 1–2 days and cultured for 5 days *in vitro*, then the cell morphology was examined by an inverted microscope. Scale bars, 0.2 μm. **(B)** Primary cortical neurons of C57BL/6J mice were stained with anti-NeuN antibody (green) and DAPI (nucleus, blue), and the ratio of cortical neurons to total cells is shown (right). Scale bars, 50 μm. 20×. Data are mean ± SD (*n* = 2).

Supplementary Figure 5 The dynamic curve of HSV-1 penetration. SH-SY5Y and N2a cells were incubated for 1 h at 4°C, then infected with HSV-1 (MOI = 20) for another 1 h at 4°C in the presence of Hsp90 inhibitors, and all groups were shifted to 37°C, incubated for different times, then washed with acidic PBS. The total HSV-1 DNA was extracted for the qRT-PCR experiment to analyze the dynamic curve of HSV-1 penetration. Data are mean ± SD (*n* = 3).

Supplementary Figure 6 Hsp90 inhibitors do not affect the total amount of cofilin. **(A,B)** Hsp90 inhibitors did not alter cofilin protein levels at the stage of HSV-1 attachment. SH-SY5Y and N2a cells were incubated for 1 h at 4°C, then infected with HSV-1 (MOI = 50) for another 1 h in the presence of 17AAG or AT533. Then the unbound virus was removed, and the protein levels of cofilin were detected by Western blot. **(C,D)** Hsp90 inhibitors did not alter cofilin protein levels at the stage of HSV-1 penetration. SH-SY5Y and N2a cells were pre-cooled for 1 h at 4°C, then infected with HSV-1 (MOI = 50) for another 1h at 4°C. The unbound virus was removed, then cells were treated with 17AAG or AT533 for indicated times at 37°C, and washed with acidic PBS to remove the viruses on cell membranes, and the protein levels were detected by Western blot. Data are mean ± SD (*n* = 3).

Supplementary Table 1 List of Primers information for qRT-PCR in this research.

Supplementary Table 2 The sequence of siRNAs used in this study.
