# Supplementary figures and images for: Hsp90 Inhibitors Inhibit the Entry of Herpes Simplex Virus 1 Into Neuron Cells by Regulating Cofilin-Mediated F-Actin Reorganization
Source: Front Microbiol. 2022 Jan 10;12:799890. doi: 10.3389/fmicb.2021.799890 (PMC8785254; doi:10.3389/fmicb.2021.799890)

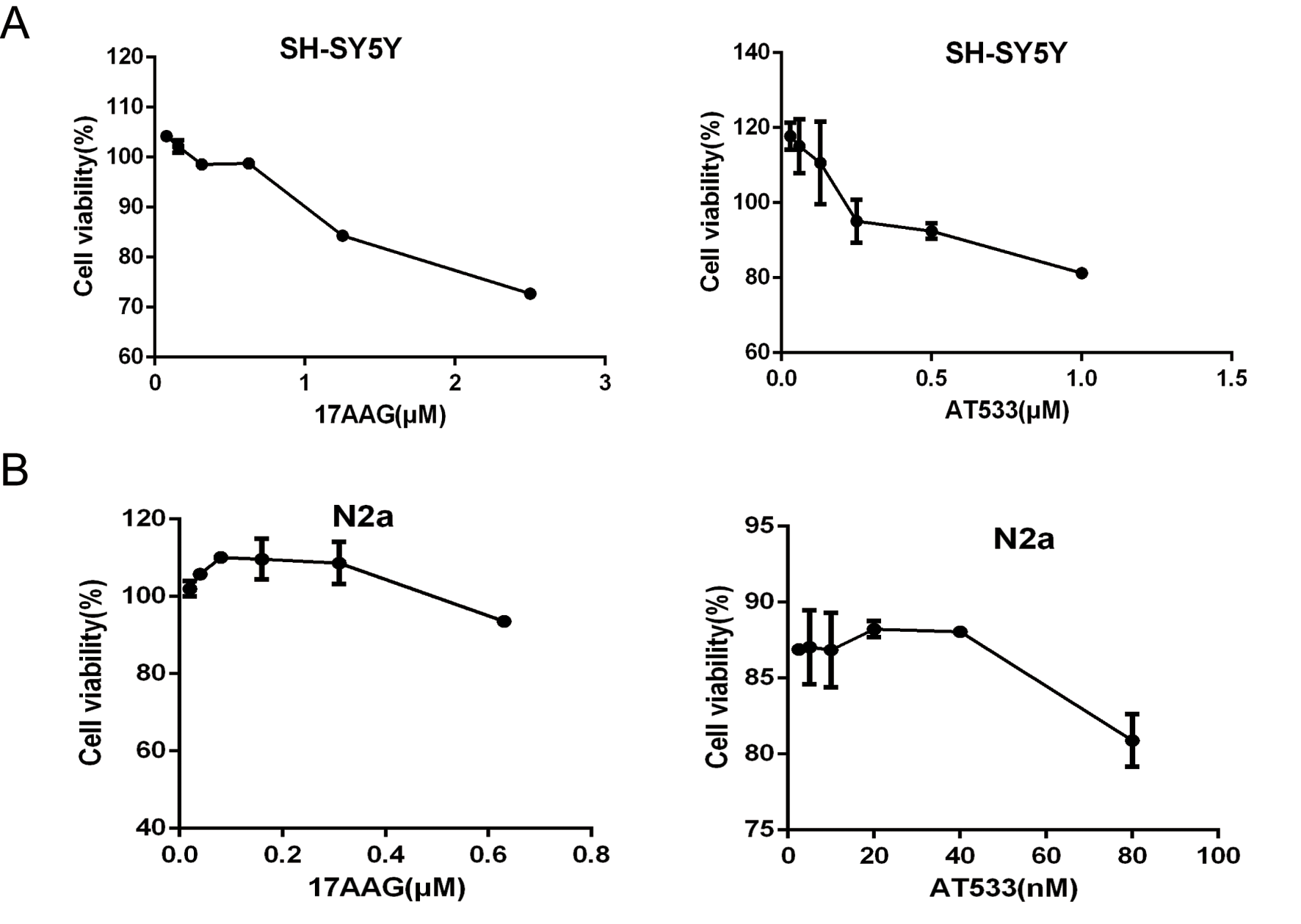

Supplement: Supplementary Figure 1 — The cytotoxicity of Hsp90 inhibitors. (A,B) The cytotoxicity of Hsp90 inhibitors. SH-SY5Y and N2a cells were treated with different concentrations of 17AAG, AT533 for 24 h, and the cell survival was then calculated through CCK8 assay. Data are mean ± SD (n = 3). [file Image_1.tif]

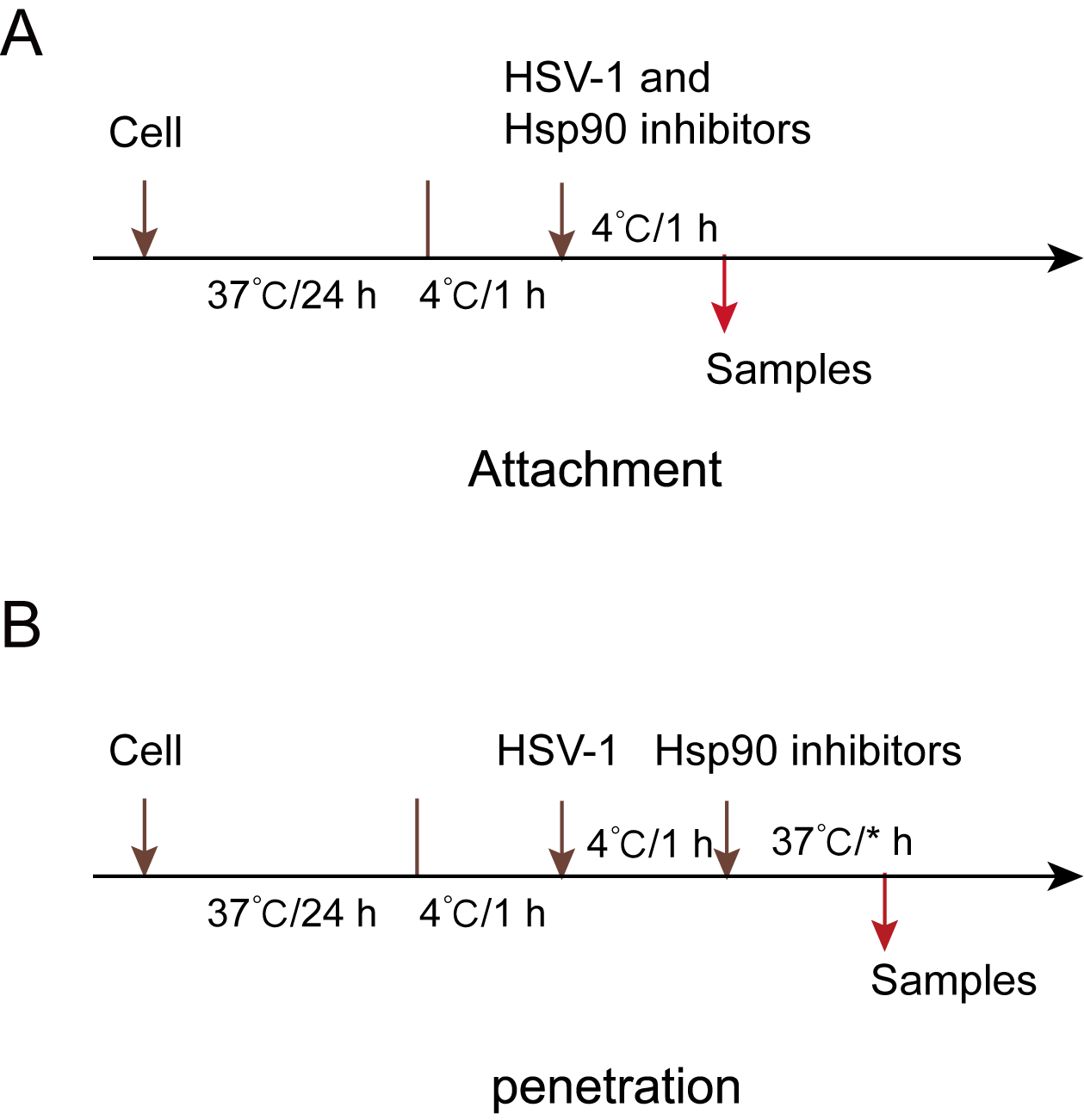

Supplement: Supplementary Figure 2 — Simple diagram of HSV-1 attachment and penetration. (A) Cells were incubated for 1 h at 4°C, then infected with HSV-1 for another 1 h in the presence of 17AAG or AT533, and the unbound virus was removed after HSV-1 attachment (B) Cells were pre-cooled for 1 h at 4°C, then infected with HSV-1 for another 1h at 4°C. The unadsorbed viruses were removed, then the cells were treated with 17AAG or AT533 for indicated times at 37°C, and washed with acidic PBS to remove the viruses on cell membranes. All samples were collected to detect and analyze. [file Image_2.tif]

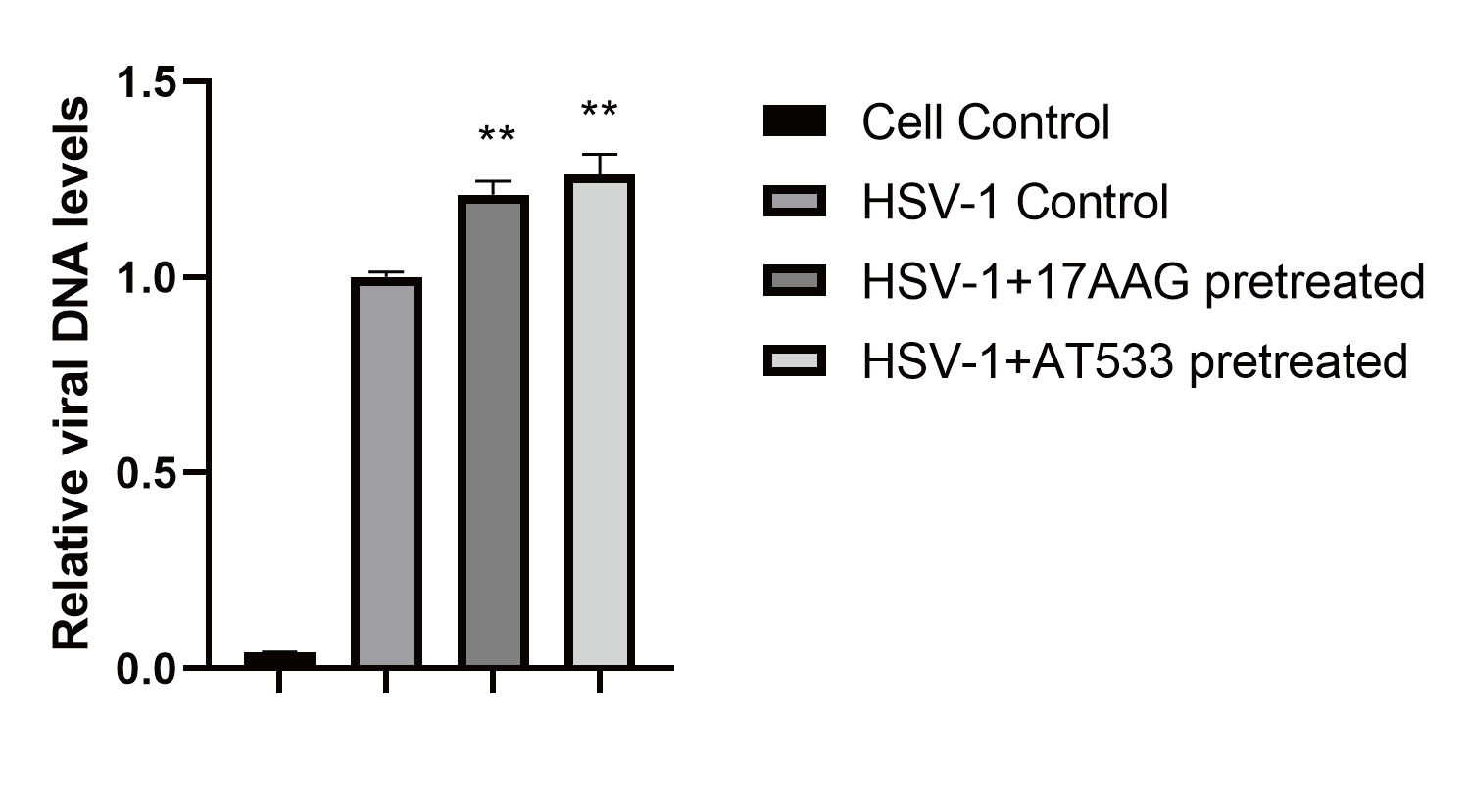

Supplement: Supplementary Figure 3 — Hsp90 inhibitors pretreatment promoted HSV-1 attachment. SH-SY5Y cells were pretreated with 17AAG or AT533 for 1 h at 4°C, and washed with PBS to remove the inhibitors, then infected with HSV-1 (MOI = 20) in the absence of 17AAG and AT533 for another 1 h. Total DNA of HSV-1 was extracted for qRT-PCR analysis to detect the level of UL47 gene. Data are mean ± SD (n = 3). **P < 0.01 versus HSV-1 control group. [file Image_3.tif]

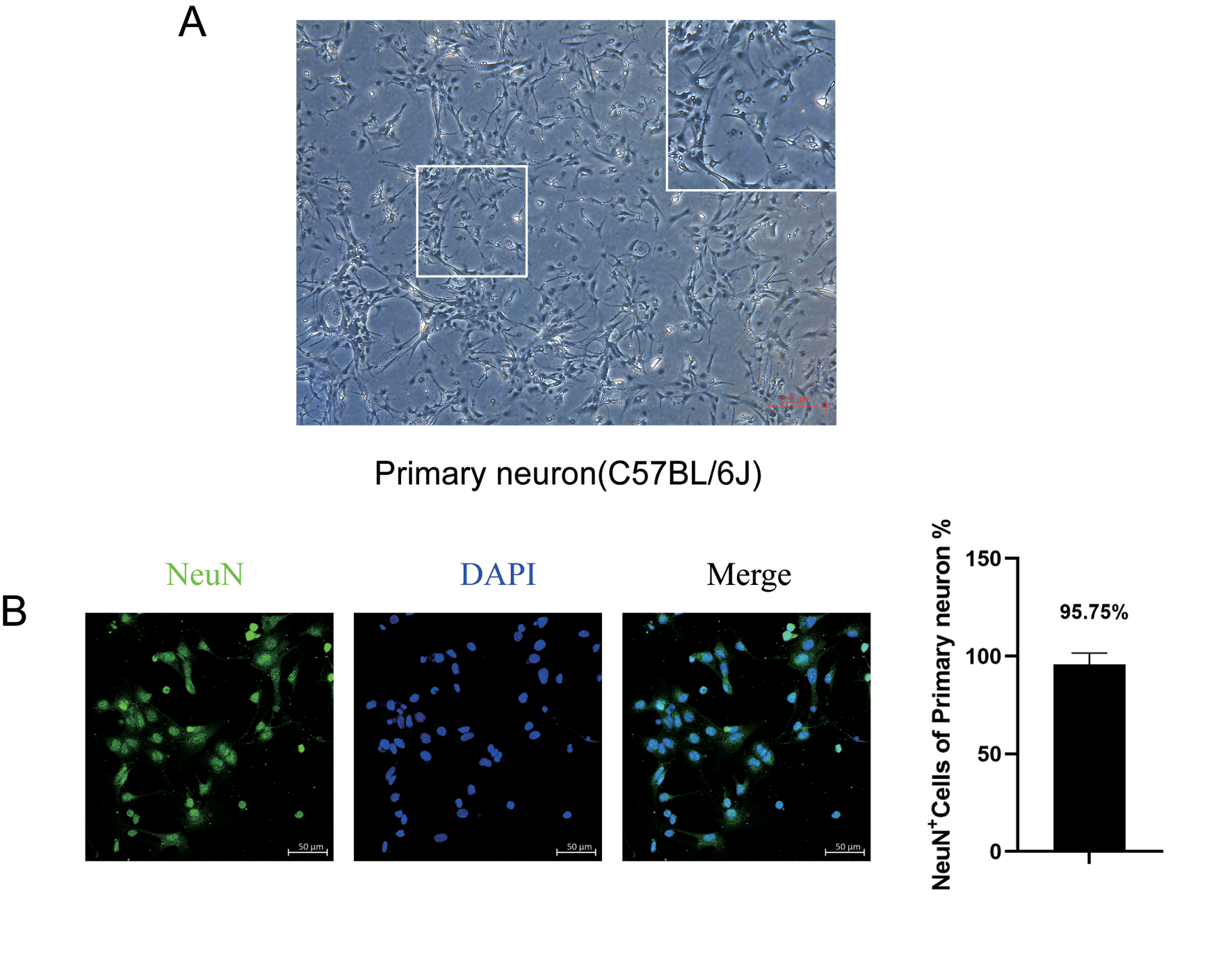

Supplement: Supplementary Figure 4 — The cell morphology of cortical neurons. (A) Primary cortical neurons were extracted from the cerebral cortex of C57BL/6J mice aged 1–2 days and cultured for 5 days in vitro, then the cell morphology was examined by an inverted microscope. Scale bars, 0.2 μm. (B) Primary cortical neurons of C57BL/6J mice were stained with anti-NeuN antibody (green) and DAPI (nucleus, blue), and the ratio of cortical neurons to total cells is shown (right). Scale bars, 50 μm. 20×. Data are mean ± SD (n = 2). [file Image_4.tif]

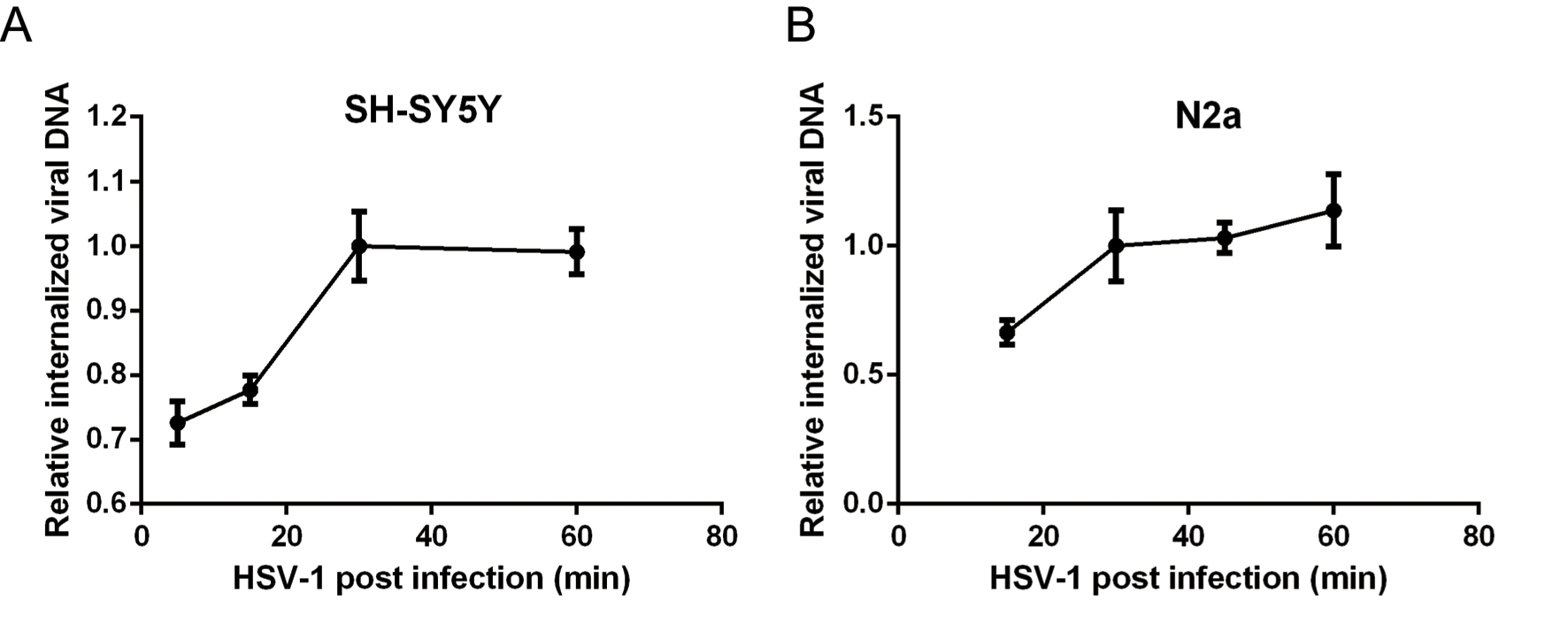

Supplement: Supplementary Figure 5 — The dynamic curve of HSV-1 penetration. SH-SY5Y and N2a cells were incubated for 1 h at 4°C, then infected with HSV-1 (MOI = 20) for another 1 h at 4°C in the presence of Hsp90 inhibitors, and all groups were shifted to 37°C, incubated for different times, then washed with acidic PBS. The total HSV-1 DNA was extracted for the qRT-PCR experiment to analyze the dynamic curve of HSV-1 penetration. Data are mean ± SD (n = 3). [file Image_5.tif]

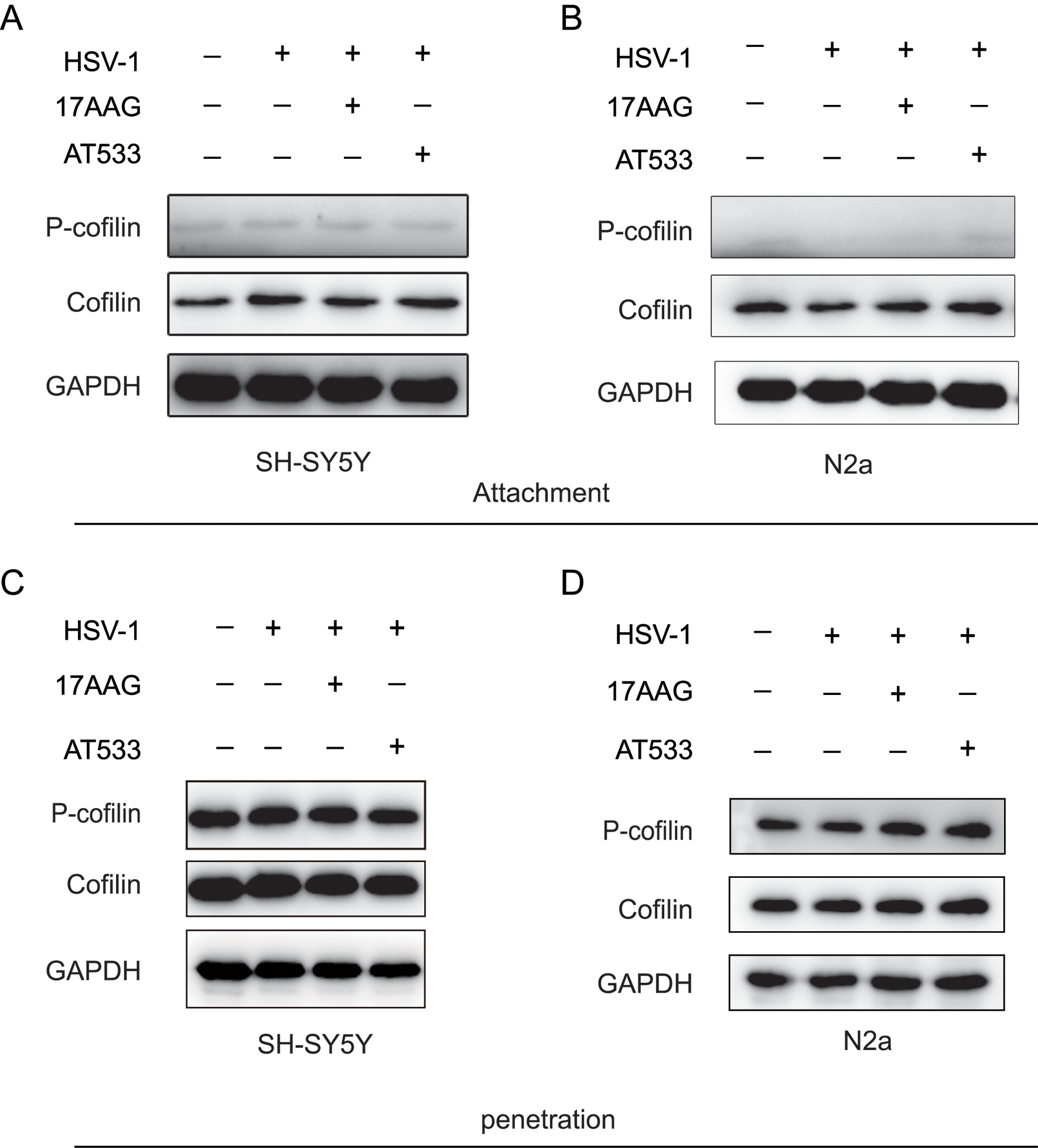

Supplement: Supplementary Figure 6 — Hsp90 inhibitors do not affect the total amount of cofilin. (A,B) Hsp90 inhibitors did not alter cofilin protein levels at the stage of HSV-1 attachment. SH-SY5Y and N2a cells were incubated for 1 h at 4°C, then infected with HSV-1 (MOI = 50) for another 1 h in the presence of 17AAG or AT533. Then the unbound virus was removed, and the protein levels of cofilin were detected by Western blot. (C,D) Hsp90 inhibitors did not alter cofilin protein levels at the stage of HSV-1 penetration. SH-SY5Y and N2a cells were pre-cooled for 1 h at 4°C, then infected with HSV-1 (MOI = 50) for another 1h at 4°C. The unbound virus was removed, then cells were treated with 17AAG or AT533 for indicated times at 37°C, and washed with acidic PBS to remove the viruses on cell membranes, and the protein levels were detected by Western blot. Data are mean ± SD (n = 3). [file Image_6.tif]

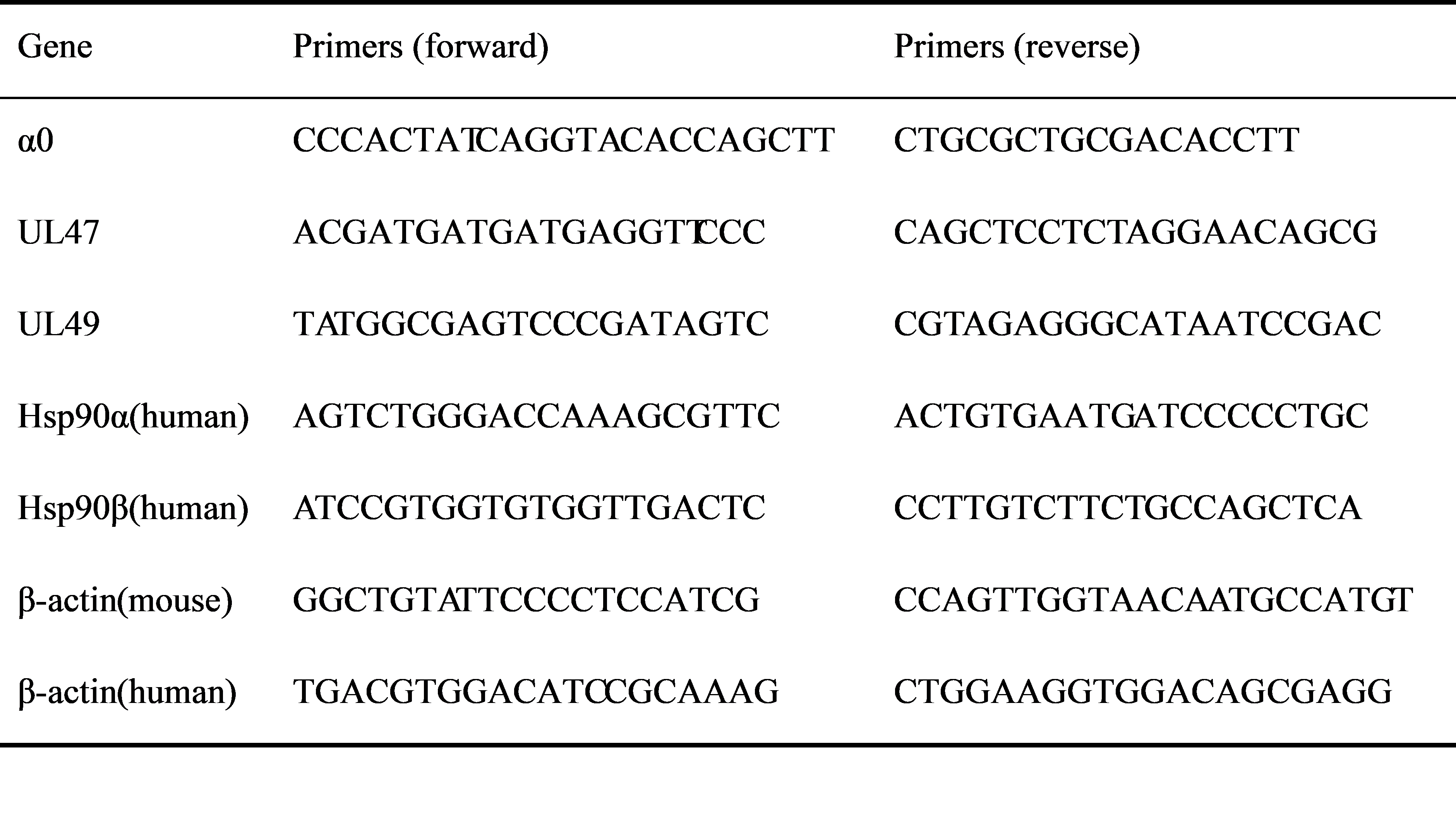

Supplement: Supplementary Table 1 — List of Primers information for qRT-PCR in this research. [file Table_1.docx]

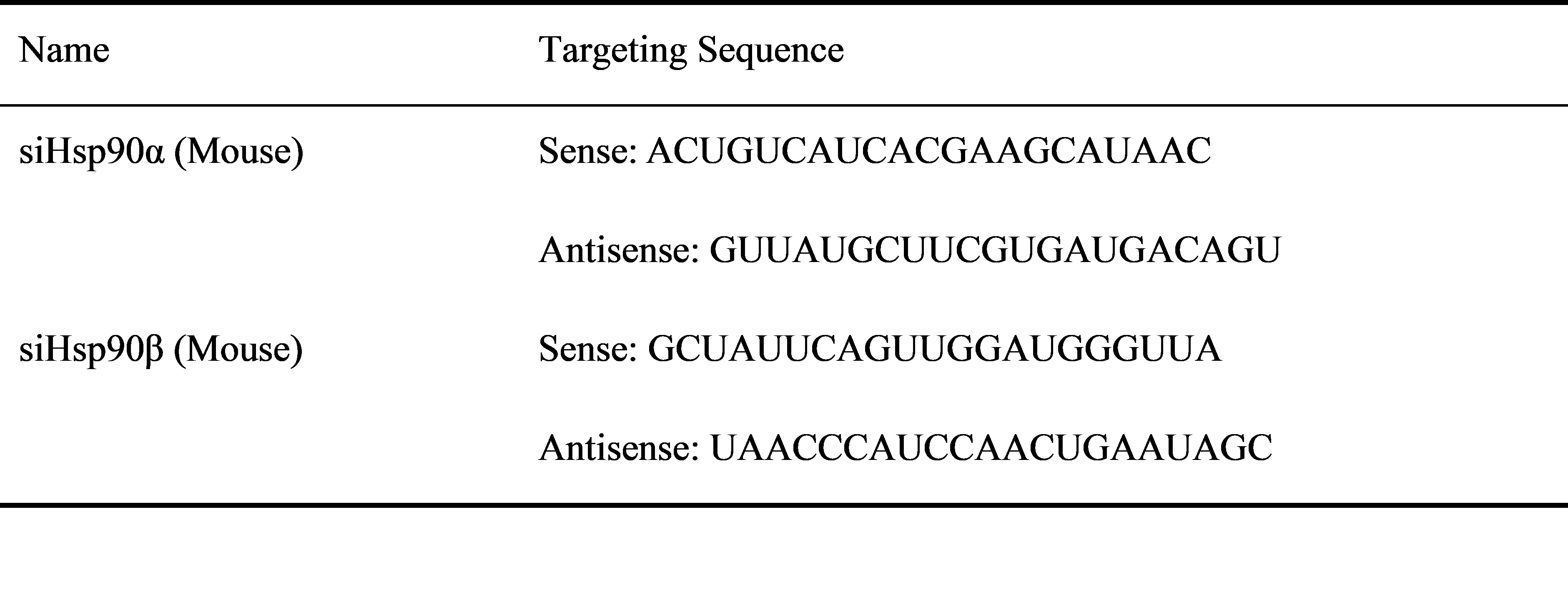

Supplement: Supplementary Table 2 — The sequence of siRNAs used in this study. [file Table_2.docx]
